# Supplementary figures and images for: Default mode network connectivity predicts individual differences in long-term forgetting: Evidence for storage degradation, not retrieval failure
Source: PLoS Comput Biol. 2025 Sep 12;21(9):e1013485. doi: 10.1371/journal.pcbi.1013485 (PMC12448966; doi:10.1371/journal.pcbi.1013485)

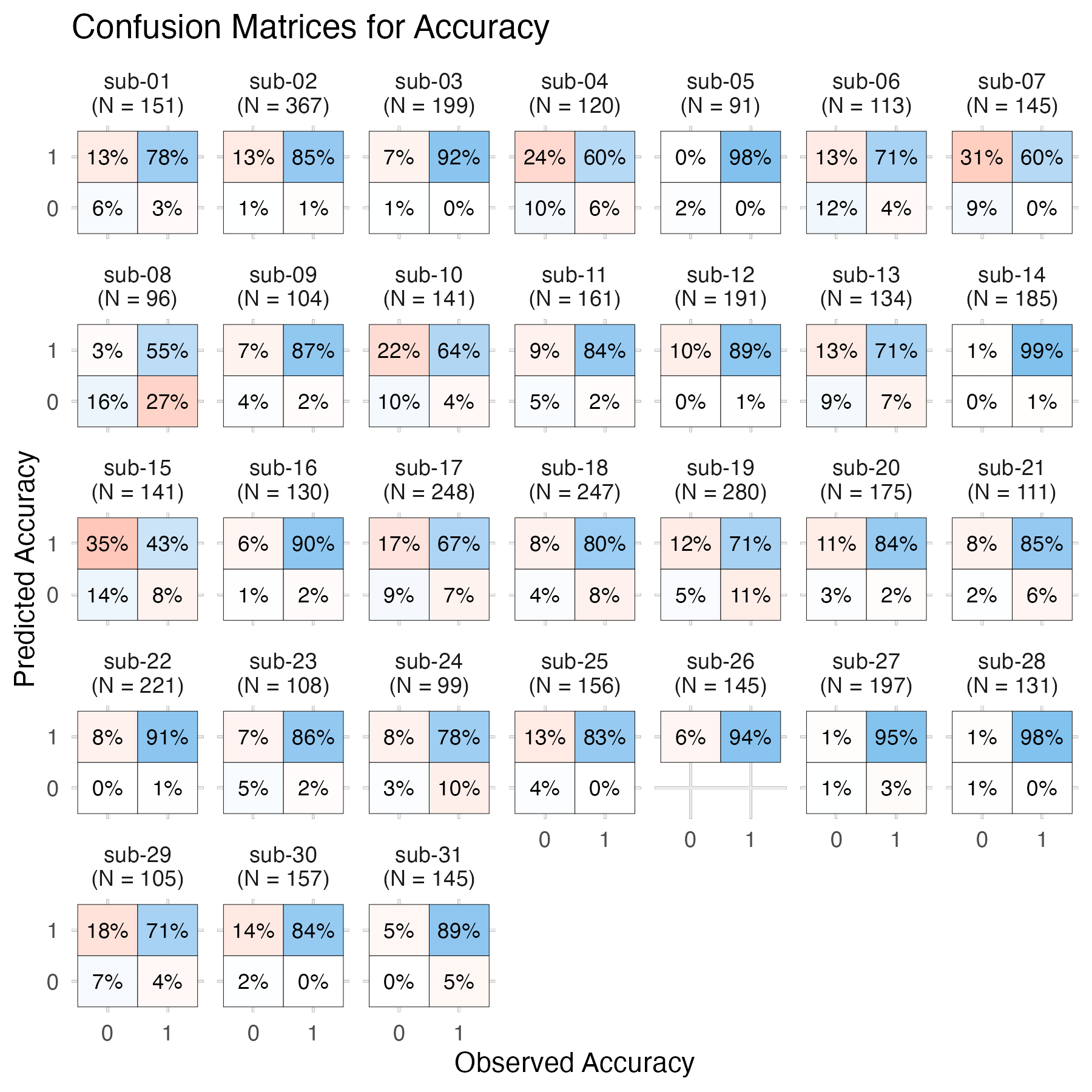

Supplement: S1 Fig — Accuracy predictions were derived from the retrieval probabilities of Eq. 3, coded as 0 (if the probability of retrieval is ≤ 0.5) and 1 (if the probability > 0.5). Numbers inside each cell indicate the percentage of trials in each category; colors indicate whether the prediction matched (blue palette) or mismatched (red palette) the observed trial accuracy; the total number of trials for each participant is indicated above each matrix. (TIF) [file pcbi.1013485.s001.tif]

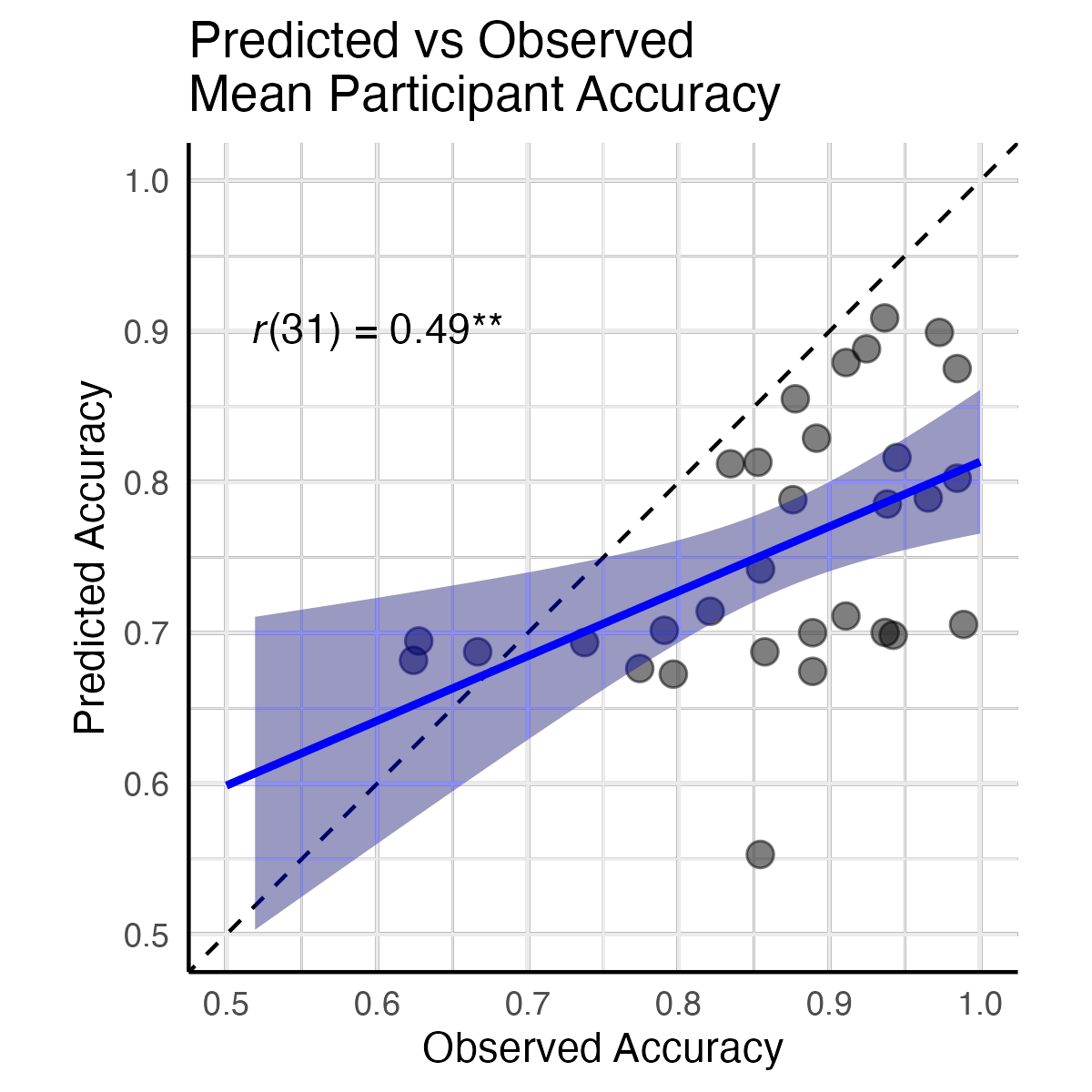

Supplement: S2 Fig — Points represent individual participants; the solid blue line represents the best-fitting regression model; the shaded area represents 95% confidence intervals of the estimate. (TIF) [file pcbi.1013485.s002.tif]

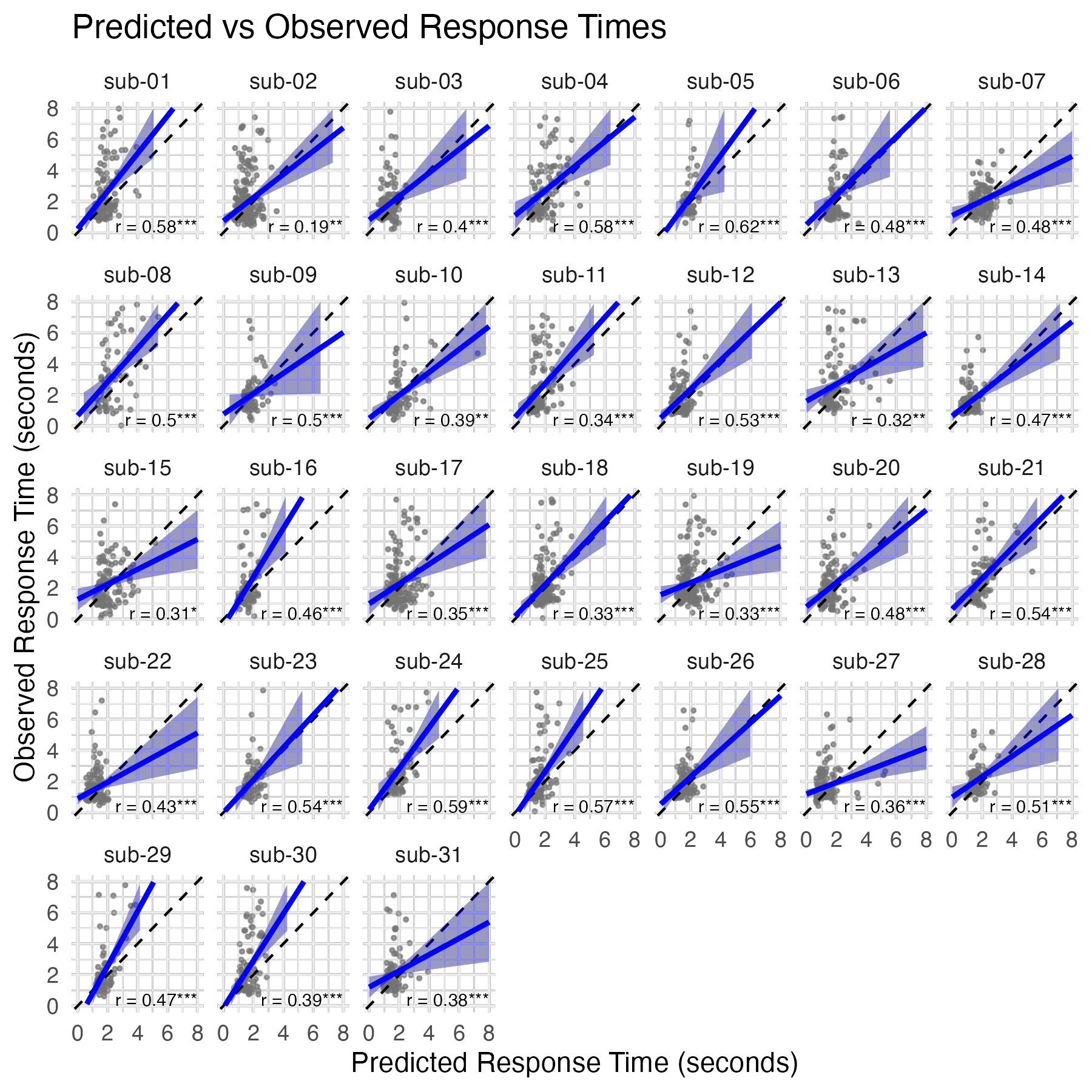

Supplement: S3 Fig — Predicted response ttimes were derived from Eq. 4. Points represent individual trials; solid lines represent the best-fitting regression lines; shaded areas represent the 95% confidence intervals. (TIF) [file pcbi.1013485.s003.tif]

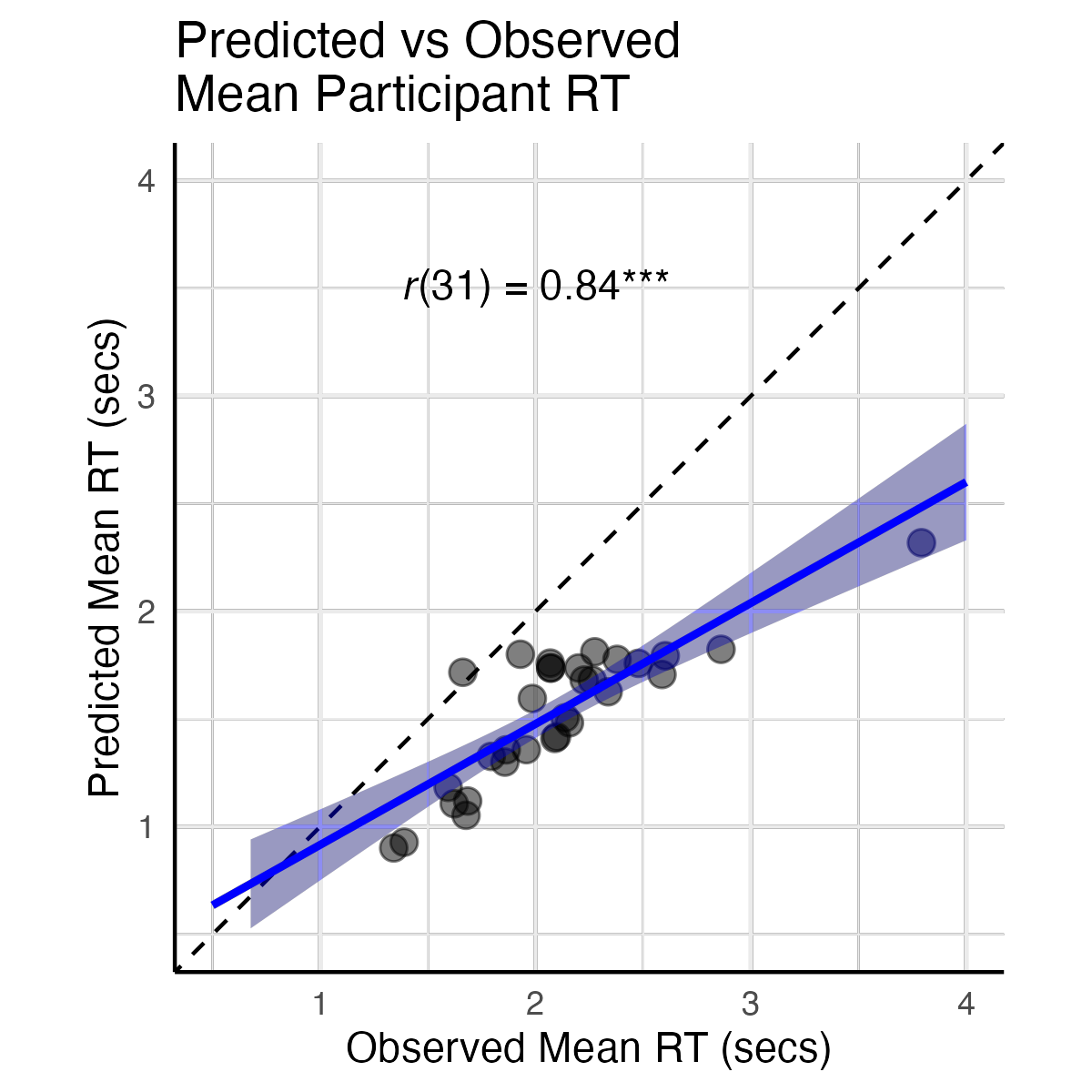

Supplement: S4 Fig — Points represent individual participants; the solid line represents the best-fitting regression line;the shaded area represent the 95% confidence interval. (TIF) [file pcbi.1013485.s004.tif]
